# Circulating Exosomal MicroRNA Profiles Associated with Acute Soft Tissue Injury

**DOI:** 10.22074/cellj.2021.7275

**Published:** 2021-08-29

**Authors:** Hongchang Yang, Jing Zhou, Junlei Wang, Luoning Zhang, Quzhi Liu, Jing Luo, Hongyan Jia, Li Liu, Qiang Zhou

**Affiliations:** 1. Physical Education Department, Hohai University, Nanjing, Jiangsu, China; 2. Department of Clinical Medicine, Jiangsu Health Vocational College, Nanjing, Jiangsu, China; 3. Harbor, Channel and Coastal Engineering, Hohai University, Nanjing, Jiangsu, China; 4. Centre of Counseling and Psychological Services, Hohai University, Nanjing, Jiangsu, China; 5. Center for Kidney Disease, 2nd Affiliated Hospital, Nanjing Medical University, Nanjing, Jiangsu, China; 6. Port Channel and Coastal Engineering Department, Hohai University, Nanjing, Jiangsu, China; 7. The Department of Rehabilitation, Brain Hospital Affiliated to Nanjing Medical University, Nanjing, Jiangsu, China

**Keywords:** Exosomes, Gene Ontology, MicroRNAs, Sequencing, Soft Tissue Injuries

## Abstract

**Objective:**

This study aimed to characterize the circulating exosomal microRNA (miRNA) profiles associated with acute soft tissue injury.

**Materials and Methods:**

In this experimental study, a total of 12 rats were randomly divided into control group and model group (n=6 for each group). The rats in the model group were used to establish an acute soft tissue injury following the mechanical injury of the leg. The exosomes from the peripheral blood of all the rats were isolated and then characterized by Nanosight NS300 particle size analyser (NTA), transmission electron microscopy (TEM) and western blot. Next, the exosomal miRNAs in the control and model groups were sequenced, and the differentially expressed miRNAs (DE-miRNAs) were identified using the DESeq algorithm. Functional analyses were performed using Gene Ontology (GO) terms and the Kyoto Encyclopedia of Genes and Genomes (KEGG) pathway databases. Finally, quantitative reverse-transcription polymersa chain reaction (qRT-PCR) was used to verify the expression of the DE-miRNAs.

**Results:**

TEM, NTA and western blot results showed that the exosomes were approximately 100 nm in size and exhibited cup-shaped morphology. A total of 628 miRNAs were obtained by sequencing. After that, 28 DE miRNAs (DE- miRNAs) were identified, including seven down-regulated miRNAs and 21 up-regulated miRNAs. These DE-miRNAs were linked to 7539 target genes with GO. Also, KEGG analyses demonstrated that these genes were enriched for phosphorylation, VEGF signaling pathway, and MAPK signaling pathway. Additionally, the consistency rate between the qRT-PCR and sequencing results was 83.33%, which showed a high relative reliability of the sequencing results.

**Conclusion:**

These findings suggest that these 28 exosomal miRNAs may be involved in the regulation of acute soft tissue injury, by one of critical biological processes (BP), phosphorylation. The findings provide valuable clues by utilizing exosomes as therapeutic targets for the effective treatment of acute soft tissue injury.

## Introduction

Acute soft tissue injury is a common clinical exercise injury and, characterized by symptoms including pain, local edema, bruising, muscle fiber breakage and limb activities disorder (1). Furthermore, this type of injury is characterized by a series of acute contusion or/and tears in the local subcutaneous soft tissue, including the acute injury of muscle, ligament, fascia, tendon, synovium, fat, joint capsule, peripheral nerves and blood vessels (2, 3). Acute soft tissue injury is usually induced by external pressure which exceeds the tissues’ threshold. This can have serious effects on people’s personal life and their career (4). Therefore, it is necessary to elucidate the molecular mechanisms of acute soft tissue injury occurrence. 

Exosomes are present in almost all biological fluids and range from 40 to 100 nm in size (5, 6). Exosomes are biologically dynamic molecules released by both healthy and damaged cells. Previous studies have reported that exosomes are a form of cellular communication that can alleviate diseases by mediating immune response or carrying messages which code for abnormal BPs (6, 7). Giudice et al. (8) reported that exosomal microRNA (miRNAs) could be applied to the differential diagnosis of bone marrow failure syndrome. MiRNAs are a class of 17-26 nt short non-coding RNAs, which control gene expression at the post transcriptional level by degrading or inhibiting of mRNA translation (6, 9). Exosome-associated miRNAs have been reported to be more stable and resistant to RNase compared with non-exosomal miRNAs (10). Additionally, miRNAs have been shown to play an important role in various physiological and pathological processes as well as in cellular homeostasis (11). Recent studies have demonstrated that miRNAs detected in body fluids including blood and urine. They are key regulatory signals for communication between cells (12, 13). Accumulating evidence suggests that some miRNAs can be abnormally expressed in the immune and cardiovascular systems after acute or chronic exercise (14, 15). Dong et al. (3) reported that hydroxysafflor yellow A, isolated from dried safflower (*C. tinctorius* L.) flowers, alleviated the increases in the expression of inflammatory cytokines (TNF-α, IL-1β, IL-6, VCAM-1, and ICAM-1) in acute soft tissue injury models. However, there is limited literature evaluating the effects of acute soft tissue injury on exosomal miRNAs profiles.

This study aimed to characterize the circulating exosomal miRNA profiles associated in an acute soft tissue injury model. These results provide valuable clues in identifying therapeutic targets for the effective treatment of acute soft tissue injuries.

## Materials and Methods

### Chemicals and reagents

In this experimental study, phosphate buffer saline (PBS) was purchased from Sangon Biotech Co., Ltd (Shanghai, China) Paraformaldehyde (4%) was obtained from China National Pharmaceutical Group Corporation (Shanghai, China). The BCA Protein Concentration Assay Kit was purchased from Boster Biological Technology Co. Ltd (Wuhan, China). RNAiso Plus (TRIzol) and PrimeScript^TM^ II 1st Strand cDNA Synthesis Kit were purchased from Takara Biomedical Technology (Beijing) Co., Ltd (Beijing, China). The cel-mir-39-3p standard RNA was obtained from Guangzhou RiboBio Co., Ltd (Guangzhou, China).

### Establishing a rat model of acute soft tissue injury

A total of 12 specific pathogen free (SPF) male Sprague Dawley (SD) rats weighing 200 ± 20 g were purchased from the Shanghai Jiesijie Experimental Animal Co., LTD (Shanghai, China). All the animals were maintained under controlled temperature (24 ± 2˚C) and humidity (50 ± 5%) conditions, with a 12- hour light/dark cycle. During the experiment, the rats had free access to food and water. After three days of acclimatization, the 12 rats were randomly divided into two equal groups: control group and model group, each of which consisted of six rats. All animal experiments were conducted in accordance with the National Medical Advisory Committee (NMAC) guidelines, using approved procedures of the Institutional Animal Care and Use Committee at Jiangsu Health Vocational College (JSJKDWLL2019001).

The previously described method for the induction of acute soft tissue injury was used here with some minor modifications (3). Firstly, all the rats were anaesthetized using an intraperitoneal injection of 2% pentobarbital sodium (75 mg/kg) and the hair of the right posterior leg was removed. An acute soft tissue injury model was established by mechanical stress. To hit the middle calf muscle of rats, a stainless steel hammer weighing 300 g was dropped from a height of 50 cm, for five consecutive times. The standard of success for these models is visible swelling and subcutaneous ecchymosis at the impact site. Any animals with fractures or skin ruptures were removed from the study. In the control group, the hair was removed while the rats were anaesthetized.

### Blood sample collection and processing

After one hour of modeling, peripheral blood (10 mL) was collected from each rat (n=3 for each group). All coagulant-free blood tubes were stored at room temperature for 30 minutes. The supernatant (approximately 5 mL) was then transferred to a sterile centrifugal tube. After placed at 4˚C for 4 hours, the sample was centrifuged at 3000 g for 15 minutes at 4˚C. After that, the supernatant was collected, and stored at -80˚C.

### Injury muscle tissue collection and histopathology analysis

After taking the blood, all the rats were killed by cervical dislocation, and injury muscle tissues were collected. The tissues were washed by phosphate buffer saline (PBS, Sangon Biotech Co., Ltd, Shanghai, China), fixed in 4% paraformaldehyde (China National Pharmaceutical Group Corporation, Shanghai, China), and then embedded in paraffin (China National Pharmaceutical Group Corporation, Shanghai, China). The 5-μm sections were cut, and stained with hematoxylin and eosin (HE) by previously described methods (16). Slides were scanned and images were taken under an optical microscope (Olympus Corporation, Tokyo, Japan).

### Isolation and characterization of exosomes

The exosome isolation procedures were performed at 4˚C and as described by Ouyang et al. (17) and Xin et al. (18). The processed plasma was thawed to room temperature, and diluted with isopycnic PBS (Sangon Biotech Co., Ltd, Shanghai, China). For eliminating dead cells and debris, the mixture was centrifuged at 1500 g for 5 minutes, and then the supernatant was transferred to a new tube. The supernatant was centrifuged again at 3500 g for 15 minutes, and then, the supernatant was transferred to another tube. After centrifuged at 10000 g for 30 minutes, the supernatant was collected, and recentrifuged at 100000 g at 4˚C for 70 minutes using an ultracentrifuge (Optima XE, Beckman Coulter, Brea, CA, USA) and the pellets were washed with pre-cooled PBS. Then, resuspension solution was recentrifuged at 100,000 g at 4˚C for another 70 minutes. The sediments were resuspended in pre-cooled PBS (200 µl) and the exosomes were isolated. The total protein concentration in the exosomes was quantitated using a BCA Protein Concentration Assay Kit (Boster Biological Technology Co. Ltd, Wuhan, China) according to the manufacturer’s instructions.

Exosomes size distribution was evaluated using a Nanosight NS300 particle size analyzer (NTA, Malvern Panalytical, Malvern, UK) as described by the method of Soares Martins et al. (19). The morphology and ultrastructure of the exosomes were visualized using transmission electron microscopy (TEM, JEOL LTD, Peabody, MA, USA), according to the method described by Zhu et al. (20). CD63, CD81 and CD9 levels, specific proteins of exosomes, were determined by western blot with their corresponding antibodies (1:1000), that contained antibodies against CD63 (Cat No. A5271, ABclonal, Boston, USA), CD81 (Cat No. ab109201, Abcam, Cambridge, UK) and CD9 (Cat No. ab92726, Abcam, Cambridge, UK) , based on the method described by Yin et al. (5).

### RNA extraction

Total RNA was isolated from exosomes using TRIzol reagent according to the manufacturer’s instructions (Takara Biomedical Technology Co., Ltd, Beijing, China). To solubilize the exosomes, 1 ml TRIzol and 25 fmol cel-mir-39-3p standards (Guangzhou RiboBio Co., Ltd, Guangzhou, China) were used. Then, Chloroform (200 µl) (China National Pharmaceutical Group Corporation, Shanghai, China) was added to separate the liquid phase, and the upper liquid phase was mixed with isopropanol (China National Pharmaceutical Group Corporation, Shanghai, China) to precipitate RNA from the exosome suspension. The sediment was then resuspended in 75% ethyl alcohol (China National Pharmaceutical Group Corporation, Shanghai, China) and centrifuged at 12,000 g and 4˚C for 5 minutes. The pellets were resuspended in 20 µl sterile water. Finally, the RNA quality was determined by measuring the 260/280 optical density (OD) ratio using a microplate reader (serial No. MK3, Thermo Fisher Scientific, Inc., Waltham, MA, USA), and 2 % agarose (Boster Biological Technology Co. Ltd, Wuhan, China) gel electrophoresis was used to check RNA integrity.

### Small RNA sequencing

Deep sequencing of exosomal small RNA profiling was performed by Yanzai biotechnology (Shanghai) Co.Ltd (Shanghai, China), as previously described (21). Burrow-Wheeler Aligner (BWA) mapping software was used to analyze the gene expression profiles. Afterwards, the miRbase database (http://www.mirbase.org/) was utilized to identify the miRNAs from the exosomes of rats. And the DESeq algorithm was applied to screen for differentially expressed miRNAs (DE-miRNAs) (log_2_ fold change (FC)>1 or <-1, false discovery rate (FDR)<0.05).

### Target genes prediction and functional analysis

Learning above mentioned DE-miRNAs roles in acute soft tissue injury, we used the Miranda and RNAhybrid algorithms (http://www.microrna.org) to predict their potential target genes (22). Gene Ontology (GO) and Kyoto Encyclopedia of Genes and Genomes (KEGG) pathway analyses were then performed by Database for Annotation, Visualization and Integrated Discovery (DAVID) tools (https://david.ncifcrf.gov/). The threshold for significantly enriched GO terms and KEGG pathways was a P<0.05. 

### Quantitative reverse-transcription polymerase chain reaction

Total RNA was used to generate cDNA via the PrimeScript™ II 1st Strand cDNA synthesis Kit (Takara Biomedical Technology Co., Ltd, Beijing, China). The primers used in the qRT-PCR reactions were designed by Primer 5.0 software based on gene sequences from the Genbank database. All primers (Table S1, See Supplementary Online Information at www.celljournal. org) were synthesized by Sangon Biotech Co., Ltd (Shanghai, China). The qRT-PCR reactions (20 µl) contained 10 µl SYBR Premix EX Taq (Takara Biomedical Technology Co., Ltd, Beijing, China), 10 nmol forward primer, 10 nmol reverse primer, 2 µl cDNA and 6 µl distilled water (Sangon Biotech Co., Ltd, Shanghai, China) . The reaction conditions were: 50˚C for 2 minutes, 95˚C for 2 minutes, 95˚C for 15 seconds and 60˚C for 60 seconds for a total of 40 cycles. The expression of cel-miR-39 was used as a standard. The relative gene expression several rno-miRs , rno-miR-122b, rno-miR-335, rno-miR-342- 3p, rno-miR-206-3p, rno-miR-215, and rno-miR-488-3p, were calculated using the 2^−ΔΔCt^ method (9).

### Statistical analysis

Graphpad prism 5 (Graphpad Software, San Diego, CA) was used to draw the graphs. And Statistical Package for Social Sciences (SPSS) Version 19.0 (SPSS Institute, Chicago, IL, USA) software was used to perform all the statistical analyses. The quantitative data were analyzed using one-way analysis of variance (ANOVA) with a post-hoc Tukey test. P<0.05 were considered statistically significant.

## Results

### Establishment of an acute soft tissue injury model

**Fig.1:**
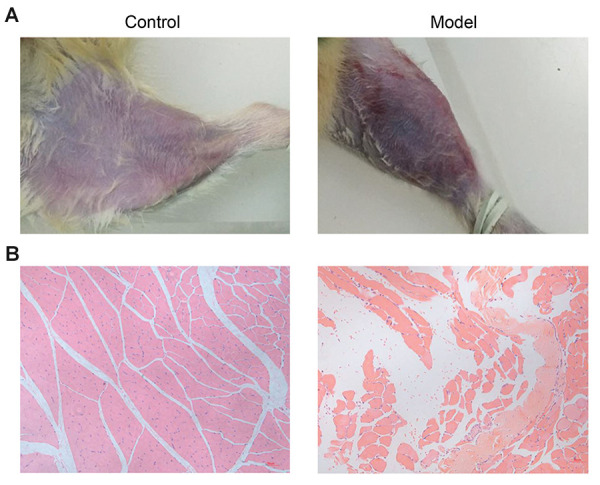
The symptoms of acute soft tissue injury in the rat model. **A.** Symptoms at a micro level. **B. **The morphology of muscle tissues was determined by Hematoxylin-Eosin (HE) staining.

Finding visible swelling and subcutaneous congestion in the model group compared to the control group, determined the successful establishment of an acute soft tissue injury model, and also, the injury degree of the impact area at a macro-level and HE staining (Fig .1A). HE staining , normal muscle morphology was observed in the control group, and severe soft tissue inflammation was seen in the model group (Fig .1B). 

### Isolation and characterization of exosomes

**Fig.2:**
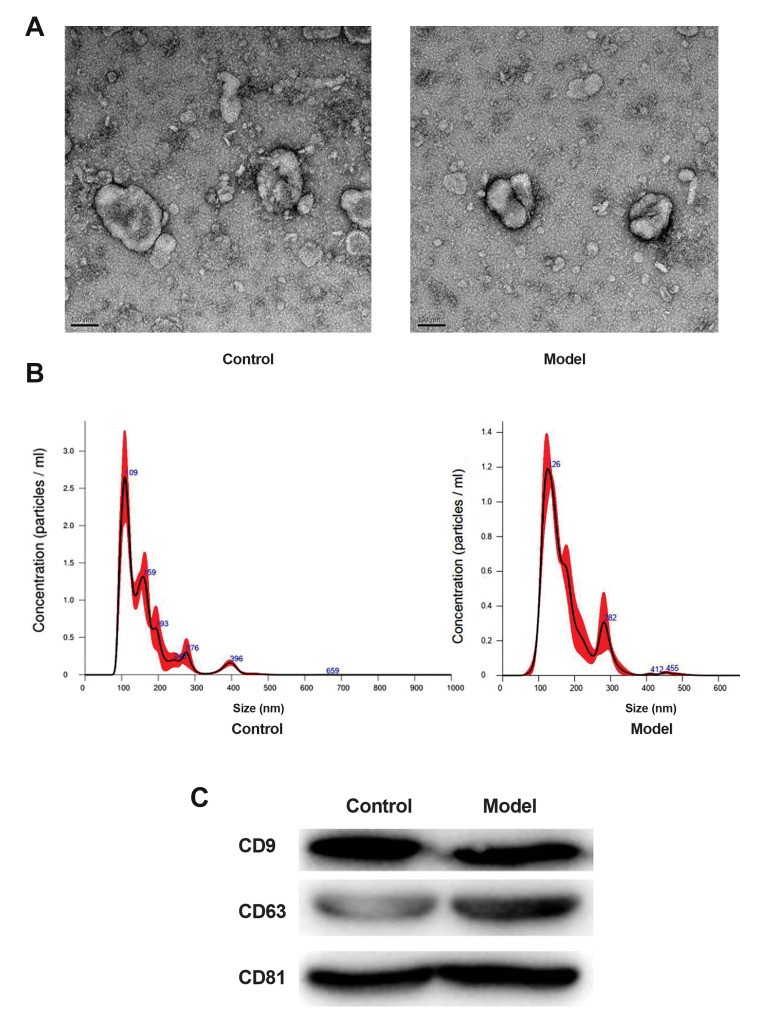
Characterization of exosomes from rat peripheral blood samples. **A. **Exosomes morphology was observed by transmission electron microscopy (TEM) (scale bar: 100 nm). **B. **Particle size distribution of exosomes was measured by Nanosight. **C.** Using western blot, exosomes surface markers (CD63, CD9, CD81) was detected.

After successful modeling, the exosomes were isolated from the peripheral blood of rats. TEM analysis , exosomes exhibited a cup-shaped or round morphology with a diameter of about 100 nm, in both groups (Fig .2A). NTA measurement indicated that the major peak in particle size was at about 109 nm or 126 nm, and the overall size distribution ranged from 100 to 200 nm (Fig .2B), which was consistent with the previously reported findings (17, 23). Additionally, exosome markers CD9, CD63 and CD81 were expressed in all samples which has been analysed by western blot analysis (Fig .2C). These results indicated this ultracentrifugation method was able to isolate exosomes.

### Exosomal miRNA expression profiles before and after soft tissue injury

**Fig.3:**
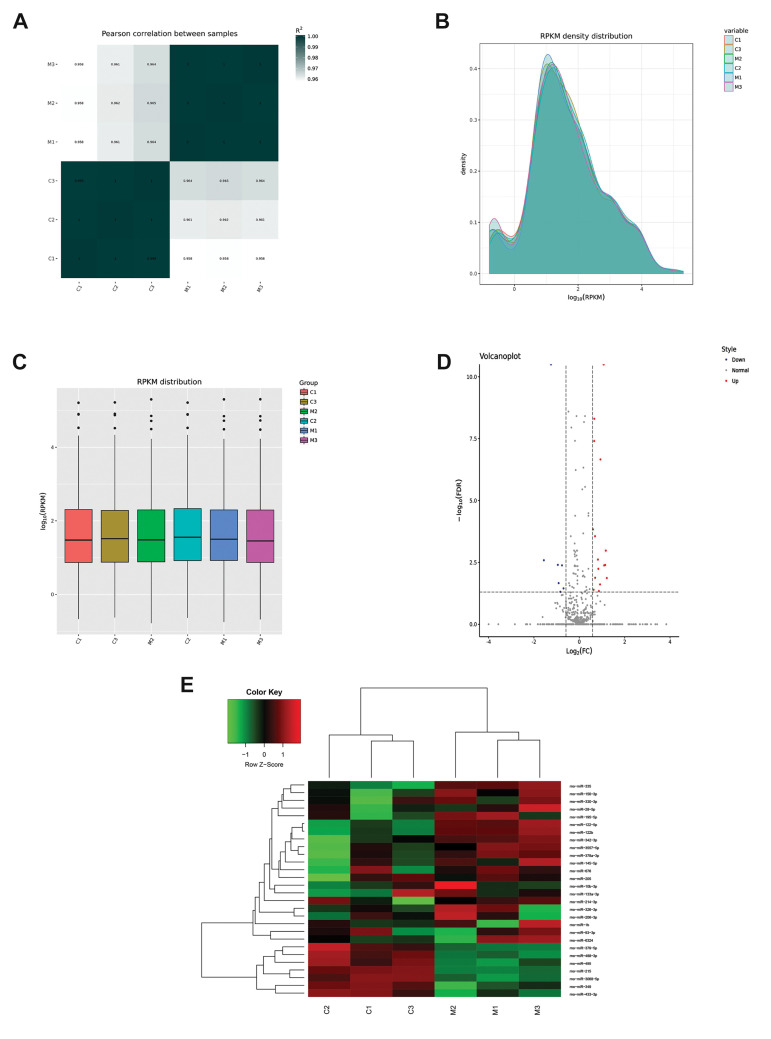
Summary of small RNA sequencing data in exosomes and screening of differentially expressed RNAs (DERs). **A.** Pearson correlation between all samples. **B.** The RPKM density distribution of all samples. **C. **The RPKM distribution of all samples. **D. **The DERs volcano diagram. The blue points represent down-regulated transcripts; the grey points represent unchanged transcripts; the red points represent the up-regulated tanscripts.** E.** Bidirectional hierarchical clustering heatmap based on the expression level of the DERs.

The differential expression of miRNAs before and after soft tissue injury was evaluated using exosome sequencing. In all groups, a range of 20, 606, 567-29, 168, 237 raw reads was obtained (Table S2, See Supplementary Online Information at www.celljournal.org). Filtering out low quality and nonsense reads, using Fast-QC (http://www. bioinformatics.babraham.ac.uk/projects/fastqc) software, about 10,000,000 clean reads (with lengths between 17 and 26 bp) were obtained for each sample (Table S2, See Supplementary Online Information at www.celljournal. org). Based on the Pearson correlation, we found no difference within the samples of each group, while there were significant differences between the control group and model group (Fig .3A). After quality control analysis, of the Reads Per Kilobase per Million mapped reads (RPKM) distribution was investigated using BWA (http://maq.sourceforge.net/bwa-man.shtml). The results showed that most of the genes were expressed and can be used for subsequent analyses (Fig .3B, C).

A total of 628 known miRNAs from the miRbase database were identified. Among them, there were 520 and 523 known miRNAs in the control and model groups, respectively. Based on the criteria, log_2_ FC>1 or <-1, and FDR<0.05, 28 DE-miRNAs were identified between this study groups, including seven down-regulated and twenty one up-regulated miRNAs (Fig .3D, Table 1). Further analysis of their hierarchical clustering showed that these 28 DE-miRNAs could easily distinguish the control and model groups, which indicated the reliability of these results (Fig .3E).

**Table 1:** Differentially expression miRNAs (DE-miRNAs) of all samples


ID	log2FC	P value	FDR	Style

rno-miR-122b	1.078827	<0.001	0	Up
rno-miR-122-5p	1.068229	<0.001	0	Up
rno-miR-215	-1.24811	<0.001	0	Down
rno-miR-335	1.068926	2.85E-39	1.11E-37	Up
rno-miR-342-3p	0.649081	3.04E-31	9.46E-30	Up
rno-miR-378a-3p	0.659442	7.07E-18	1.47E-16	Up
rno-miR-3557-5p	0.599633	1.96E-14	3.04E-13	Up
rno-miR-150-3p	0.659581	4.17E-10	4.99E-09	Up
rno-miR-145-5p	0.6574	3.82E-09	3.95E-08	Up
rno-miR-676	0.931966	2.18E-08	2.19E-07	Up
rno-miR-330-3p	0.606893	1.83E-05	0.000146	Up
rno-miR-214-3p	0.692557	3.68E-05	0.000276	Up
rno-miR-206-3p	1.169269	0.00016	0.00106	Up
rno-miR-326-3p	0.8205	0.000409	0.002446	Up
rno-miR-3068-5p	-1.56329	0.00045	0.002591	Down
rno-miR-488-3p	-0.94457	0.000705	0.003987	Down
rno-miR-205	1.141366	0.000726	0.004029	Up
rno-miR-1b	1.096268	0.000779	0.004174	Up
rno-miR-379-5p	-0.76485	0.000769	0.004174	Down
rno-miR-10b-3p	0.839152	0.001185	0.00576	Up
rno-miR-6324	0.697845	0.003149	0.013234	Up
rno-miR-195-5p	1.21186	0.003254	0.013494	Up
rno-miR-349	-0.9123	0.005391	0.021719	Down
rno-miR-28-5p	0.915729	0.006312	0.024539	Up
rno-miR-495	-0.69833	0.0097	0.03549	Down
rno-miR-133a-3p	0.631021	0.011299	0.040391	Up
rno-miR-93-3p	0.863672	0.013013	0.045474	Up
rno-miR-433-3p	-0.83785	0.014262	0.048741	Down


FC; Fold change, FDR; False discovery rate, Up; Up-regulation, and down; Down-regulation.

### Prediction of target genes and functional analysis

**Fig.4:**
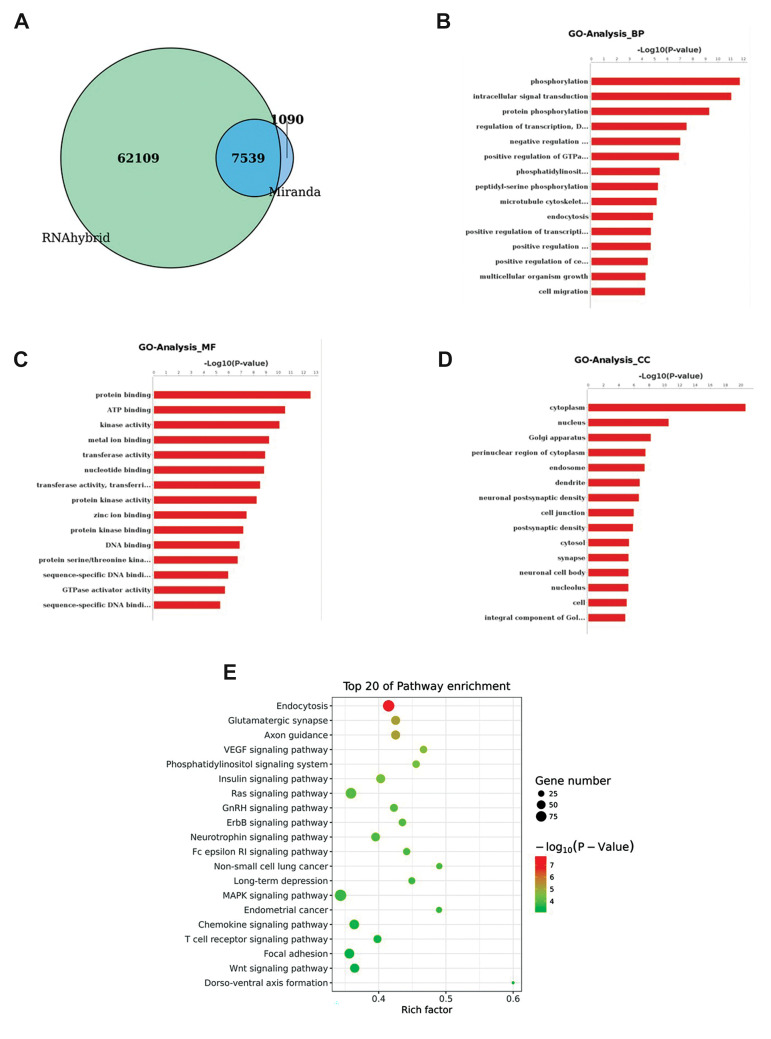
Functional annotation of the potential target genes of DE-miRNAs. **A. **The predicted target genes identified using the Miranda and RNAhybrid algorithms.** B-D. **The GO analysis performed using DAVID. **E. **The signaling pathway analysis carried out using the KEGG database. DE; Differentially expressed, GO; Gene ontology, and KEGG; Kyoto encyclopedia of genes and genomes.

A total of 7539 target genes were predicted using the Miranda and RNAhybrid algorithms for the 28 DE-miRNAs (Fig .4A). GO term analysis was then performed on these target genes. Figure 4B-D showed the most enriched GO terms in BP, molecular function (MF) and cellular component (CC). In BP analysis, phosphorylation, intracellular signal transduction, and protein phosphorylation were found to be associated with acute soft tissue injury. Besides, protein binding and ATP binding were found to be the most enriched GO terms in MF analysis. And in CC analysis, most of the miRNAs were proved to be related to the cytoplasm. KEGG pathway analysis was also applied to investigate the signaling pathways of these target genes. The most commonly represented signaling pathways for these genes were endocytosis, VEGF signaling pathway, phosphatidylinositol signaling system and MAPK signaling pathway (Fig .4E). 

### Quantitative reverse-transcription polymerase chain reaction validation

**Fig.5:**
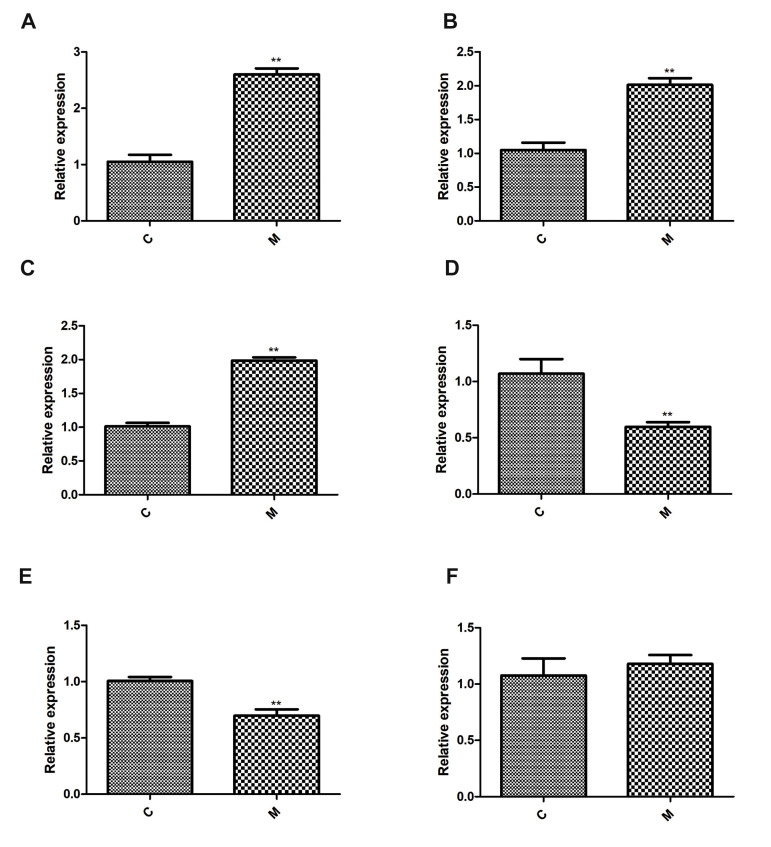
Validation of relative expression of miRNAs by qRT-PCR. **A. **miR-122b, **B.** miR-335, **C.** miR-206-3p, **D. **miR-215, **E.** miR-488-3p, and **F. **miR-342-3p. **; P<0.01 vs. the control group and qRT-PCR; Quantitative reverse-transcription polymerase chain reaction.

Four up-regulated miRNAs (rno-miR-122b, rno-miR-335, rno-miR-342-3p, rno-miR-206-3p) and two down-regulated miRNAs (rno-miR-215, rno-miR-488- 3p) were chosen for qRT-PCR analysis to validate the sequencing results. Based on the results of the qRT-PCR analysis, we found that the expressions of rno-miR-122b, rno-miR-335, and rno-miR-206-3p were all up-regulated, while the expressions of rno-miR-215 and rno-miR-488- 3p were down-regulated compared to control group, which showed a consistent expression pattern with those suggested by the sequencing results (Fig .5A-E). The expression level of miR-342-3p was not significantly different between groups (Fig .5F). We found 83.33% consistency rate for the qRT-PCR and the sequencing data, which reveals a high degree of reliability of the sequencing data.

## Discussion

Acute soft tissue injury leads to nonspecific physiological responses that activate a series of proinflammatory events and accounts for the majority of work in emergency departments (24). The main pathological changes of acute soft tissue injury are traumatic aseptic inflammation, manifested as telangiectasia, local tissue necrosis, edema and bleeding. In recent years, we have seen an increase in the number of studies using a blunt trauma-induced acute soft tissue injury model. In this study, we established a rat model of acute soft tissue injury using a self-made hammer. Our results provide a basis for the elucidation of the internal mechanism underlying exosome associated miRNA-mediated regulation of acute soft tissue injury.

Increasing evidence suggests that miRNAs play critical regulatory roles in many biological activities, such as cellular proliferation, differentiation, migration and disease progression (25, 26). However, the roles of exosome associated miRNAs in acute soft tissue injury remain unclear. Our findings for the first time have identified 28 DE-miRNAs, including seven down-regulated miRNAs and 21 up-regulated miRNAs that may be important to acute soft tissue injury development or improvement. Among these DE-miRNAs, miR-206-3p, miR-378a-3p and miR-133a-3p have been reported to be associated with both slow and fast muscle damage (27). Our results showed that the expression levels of miR-206- 3p, miR-378a-3p and miR-133a-3p were all up-regulated after soft tissue injury. A study of Tang et al. (28) indicated that miR-206-3p decreased the phosphorylation of Akt, which is the downstream effector of c-Met in the PI3K/ Akt signaling pathway. Additionally, the high expression levels of miR-378a-3p and miR-133a-3p were reported to reflect the inflammatory levels (29, 30). Combined with our results, we speculated that the high expression levels of miR-206-3p, miR-378a-3p and miR-133a-3p may promote the development of acute soft tissue injury. 

Previous studies have shown that miR-335 and miR-488-3p, promising biomarkers of the traumatic brain injury (31, 32), were up-regulated and down-regulated in the pro-inflammatory environment, respectively (33, 34), which was consistent with that in our study. Moreover, our results showed that miR-342-3p, miR-122-5p and miR-215 were all up-regulated in our acute soft tissue injury. And VEGF signaling pathway and MAPK signaling pathway were mediated by these miRNAs downstream target genes using KEGG. In previous researches, miR-342-3p, miR-122-5p and miR-215 were found to be involved in the inflammatory responses of different diseases (35-37). Furthermore, the VEGF signaling pathway was involved in the generation and formation of blood vessels . A study of Li et al. (38) showed that emodin alleviated LPS-induced inflammation through inhibiting mTOR/HIF-1α/ VEGF signaling pathway. MAPK signaling pathway can regulate cell growth, differentiation, the adaptive ability to environmental stress, inflammatory responses and other important cellular physiological/ pathological processes (39). Based on the all these findings, it was speculated that miR-335, miR-488-3p, miR-342-3p, miR-122-5p and miR-215 may be potential biomarkers of acute soft tissue injuries, and VEGF signaling pathway and MAPK signaling pathway may be involved in the inflammatory response to acute soft tissue injuries. However, the roles of the other DE-miRNAs in acute soft tissue injuries remain unclear, therefore the function and molecular mechanisms of exosomes associated miRNAs need to be studied in more detail at a later data.

These 28 DE-miRNAs identified in the exosomes were predicted to target 7539 downstream genes. GO analysis of these genes showed that the phosphorylation in BP and protein binding in MF were enriched in acute soft tissue injury. Jin et al. (40) shown that traumatic brain injury significantly increased the phosphorylation of glial gap junction protein connexin 43 (Cx43) and promoted the release of exosomes, which was consistent with our results. Additionally, Dong et al. (3) indicated that hydroxysafflor yellow A decreases p38 MAPK phosphorylation in skeletal muscle, and inhibits the expression of pro-inflammatory cytokines, thus attenuats acute soft tissue injury. When these studies are combined with our results, phosphorylation may be the key biological process in the propagation of acute soft tissue injury responses.

However, there are still some limitations to this study. First, a larger sample size should be desirable. Considering human samples would help to remove any model discrepancies. Individual analysis of the DE-miRNAs should be carried out to identify and understand the underlying biological mechanisms affecting this type of injury. Importantly, the specific functions of the identified exosomal miRNAs in acute soft tissue injury will be further explored utilizing *in vivo*.

## Conclusion

In this study, our findings help to identify targets for the development of novel therapeutic interventions. These results indicated that these 28 DE-miRNAs may be a potential biomarker for acute soft tissue injury and phosphorylation may be the key biological process in the development of acute soft tissue injury.

## Supplementary PDF


