# Supplementary material for: Circulating Exosomal MicroRNA Profiles Associated with Acute Soft Tissue Injury
Source: Cell J. 2021 Aug 29;23(4):474–84. doi: 10.22074/cellj.2021.7275 (PMC8405084; doi:10.22074/cellj.2021.7275)
Supplement: Supplementary file 1 [file Cell-J-23-474-s01.pdf]

Supplementary Information for

Circulating Exosomal MicroRNA Profiles Associated with Acute Soft Tissue Injury

Hongchang Yang, M.M.[1#], Jing Zhou, M.M.[2#], Junlei Wang, B.M.[3], Luoning Zhang, B.M.[1], Quzhi Liu, Ph.D.[4], Jing Luo, M.M.[5], Hongyan Jia, B.M.[6], Li Liu, B.M.[7*], Qiang Zhou, M.M.[1*]

1. Physical Education Department, Hohai University, Nanjing, Jiangsu, China
2. Department of Clinical Medicine, Jiangsu Health Vocational College, Nanjing, Jiangsu, China
3. Harbor, Channel and Coastal Engineering, Hohai University, Nanjing, Jiangsu, China
4. Centre of Counseling and Psychological Services, Hohai University, Nanjing, Jiangsu, China
5. Center for Kidney Disease, 2nd Affiliated Hospital, Nanjing Medical University, Nanjing, Jiangsu, China
6. Port Channel and Coastal Engineering Department, Hohai University, Nanjing, Jiangsu, China
7. The Department of Rehabilitation, Brain Hospital Affiliated to Nanjing Medical University, Nanjing, Jiangsu, China

#These authors contributed equally to this work.

**Corresponding Addresses: The Department of Rehabilitation, Brain Hospital Affiliated to Nanjing Medical University, Nanjing, Jiangsu, China*
Physical Education Department, Hohai University, Nanjing, Jiangsu, China
Emails: liulicao1976@163.com, 19870073@hhu.edu.cn

Table S1: The primer sequences of all primers

Name	Primer sequence (5´-3´)
Common downstream primer of the neck ring method	GTGCAGGGTCCGAGGT
rno-miR-122b-RT	GTCGTATCCAGTGCAGGGTCCGAGGTATTCGCACTGGATACGACTGGAGT
rno-miR-122b-F	GCAACACCATTGTCAC
rno-miR-335-RT	GTCGTATCCAGTGCAGGGTCCGAGGTATTCGCACTGGATACGACACATTT
rno-miR-335-F	TCAAGAGCAATAACGAA
rno-miR-342-3p-RT	GTCGTATCCAGTGCAGGGTCCGAGGTATTCGCACTGGATACGACACGGGT
rno-miR-342-3p-F	TCTCACACAGAAATCGC
rno-miR-206-3p-RT	GTCGTATCCAGTGCAGGGTCCGAGGTATTCGCACTGGATACGACCCACAC
rno-miR-206-3p-F	TGGAATGTAAGGAAGT
rno-miR-215-RT	GTCGTATCCAGTGCAGGGTCCGAGGTATTCGCACTGGATACGACTGTCTG
rno-miR-215-F	ATGACCTATGATTTGA
rno-miR-488-3p-RT	GTCGTATCCAGTGCAGGGTCCGAGGTATTCGCACTGGATACGACGACCAA
rno-miR-488-3p-F	TTGAAAGGCTGTTTC
cel-miR-39-RT	GTCGTATCCAGTGCAGGGTCCGAGGTATTCGCACTGGATACGACCAAGCT
cel-miR-39-F	GGCCTCACCGGGTGTAAATCAG

Table S2: Summary of data generated from sequencing

Data type	Control group			Model group		
	1	2	3	1	2	3
Raw reads	22, 823, 864	20, 606, 567	21, 331, 721	26, 916, 892	29, 168, 237	23, 452, 792
Clean reads	9, 641, 106	8, 948, 992	8, 795, 536	11, 212, 872	12, 055, 080	9, 500, 799
